# Influence of N-protonation on electronic properties of acridine derivatives by quantum crystallography†

**DOI:** 10.1039/d3ra08081a

**Published:** 2024-02-12

**Authors:** Sylwia Pawlędzio, Marcin Ziemniak, Damian Trzybiński, Mihails Arhangelskis, Anna Makal, Krzysztof Woźniak

**Affiliations:** a Neutron Scattering Division, Oak Ridge National Laboratory Oak Ridge TN 37831 USA pawledzios@ornl.gov; b Department of Chemistry, Biological and Chemical Research Centre, University of Warsaw Żwirki i Wigury 101 02-093 Warszawa Poland kwozniak@chem.uw.edu.pl

## Abstract

Applications of 9-aminoacridine (9aa) and its derivatives span fields such as chemistry, biology, and medicine, including anticancer and antimicrobial activities. Protonation of such molecules can alter their bioavailability as weakly basic drugs like aminoacridines exhibit reduced solubility at high pH levels potentially limiting their effectiveness in patients with elevated gastric pH. In this study, we analyse the influence of protonation on the electronic characteristics of the molecular organic crystals of 9-aminoacridine. The application of quantum crystallography, including aspherical atom refinement, has enriched the depiction of electron density in the studied systems and non-covalent interactions, providing more details than previous studies. Our experimental results, combined with a topological analysis of the electron density and its Laplacian, provided detailed descriptions of how protonation changes the electron density distribution around the amine group and water molecule, concurrently decreasing the electron density at bond critical points of N/O–H bonds. Protonation also alters the molecular architecture of the systems under investigation. This is reflected in different proportions of the N⋯H and O⋯H intermolecular contacts for the neutral and protonated forms. Periodic DFT calculations of the cohesive energies of the crystal lattice, as well as computed interaction energies between molecules in the crystal, confirm that protonation stabilises the crystal structure due to a positive synergy between strong halogen and hydrogen bonds. Our findings highlight the potential of quantum crystallography in predicting crystal structure properties and point to its possible applications in developing new formulations for poorly soluble drugs.

## Abstract 2

Protonation changes the molecular architecture of the crystal structures, and thus modifies the intermolecular interactions in the studied structures.

## Graphical abstract

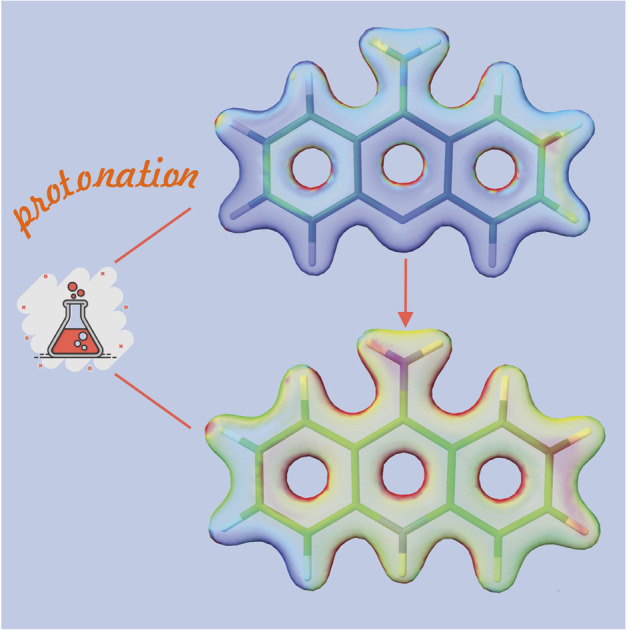

## 1. Introduction

Acridines are a class of heterocyclic compounds composed of a tricyclic aromatic system being a derivative of anthracene with one central CH group replaced by a nitrogen atom. Acridine-based derivatives have found several applications in the fields of chemistry, biology, and medicine such as fluorescent dyes,^1^ drugs^2^ and catalysts in organic synthesis.^3,4^ Despite being generally toxic and carcinogenic in mammals, 9-aminoacridine (9aa) has found several applications in medicinal chemistry, since it provides a scaffold for synthesizing several molecules, which display promising anticancer and antimicrobial activities.^3^ In living cells, aminoacridines mainly act by DNA intercalation^5,6^ and inhibition of topoisomerase II,^7^ disrupting DNA replication and, thereby, cell division making them useful antimalarial^8^ and anticancer drugs.^9^ Since 9aa derivatives often display specificity against particular types of cancer cells their mechanisms of action are not merely limited to DNA damage and it is believed that it also includes the disruption of several signalling pathways including PI3K/AKT/mTOR, p53 and NF-kappaB^10^ as well as induction of apoptosis.^11^ Interestingly, the antimalarial activity of acridines is also a complex phenomenon involving inhibition of topoisomerase II, mitochondrial proteins and formation of hemozoin, a by-product of haemoglobin decomposition crucial for heme detoxification in *Plasmodium*.^12,13^ Due to their potential efficacy against drug-resistant strains of *Plasmodium* sp., which are becoming more prevalent than in previous years, interest in acridines and their derivatives has also increased.^8,13^

The structural chemistry of acridines is quite well described.^14–16^ Most of the reported structures of 9aa are multi-component systems containing other chemical entities in their crystal lattices, often inorganic anions or solvent molecules.^17–21^ Acridine moieties are usually protonated, and intricate patterns of hydrogen bonding stabilise their crystal structures. It is known that 9aa can form different crystal structures depending on the type of ions and/or solvent molecule(s) present in the crystals. Monohydrates of 9aa halides form π-stacked columns along the *Y*(*b*)-direction which are linked *via* a N–H⋯O hydrogen bond between the endocyclic N-atom of the acridinium ring and a water molecule to produce a complex 3D architecture.^20^ These structures usually contain an R^2^_2_(8) structural motif consisting of two molecules (9aa and halogen anions, or a water moiety and a halogen anion) related by a twofold rotation axis and held together by eight hydrogen bonds. Bis-hydrated 9aa halides display similar structural features. However, their complexity is even higher since their crystals contain several different supramolecular rings.^20^ The structure of the hemihydrate of 9aa is also known and is composed of supramolecular tetramers of 9aa molecules connected *via* water-bridged hydrogen bonds, and no stacking interactions are present in the crystal.

It is well known that due to changes in its bioavailability upon protonation, the protonated form of a drug may have different pharmaceutical potency than the neutral form.^22^ In many cases, only a drug's non-protonated form can readily penetrate cell membranes when active transport is not involved.^23^ On the other hand, many weakly basic drugs, including aminoacridines, have reduced solubility at higher pH. This may become a significant issue in patients with achlorhydria or other conditions leading to high gastric pH, which could reduce the bioavailability of these drugs.^24,25^ The reduced absorption of weakly basic drugs under high gastric pH can be mitigated by several strategies including pre-treatment with organic acids,^26^ or the development of formulations containing either acidic salts or solid dosage formulations, in which an acid acts as pH modifying factor enhancing the bioavailability in the stomach.^24^ The last strategy is promising due to its simplicity and is under clinical investigation.^27^

The dynamic development of technology, X-ray diffraction equipment and dedicated computer software have made X-ray structural analysis of single crystals the most important method for determining the structure of chemical compounds. Thousands of structures are deposited annually in the CSD (Cambridge Structural Database). Determining the structure of a molecule with pharmaceutical properties (API, Active Pharmaceutical Ingredient) is linked to studies of its electronic properties and the intermolecular interactions. Aspherical atom refinement is a quantum crystallographic method used to refine crystal structures with a more accurate description of the electron density (ED) distribution than other methods.^28–31^ In traditional refinement methods such as the Independent Atom Model (IAM), atomic EDs are modelled as spherical, which is a crude approximation of the real distribution of the ED in the crystalline solid state. Aspherical atom refinement considers the non-spherical nature of the electron density distribution around atoms providing more accurate structural parameters, displacement parameters, and hydrogen-bond lengths compared to traditional refinement methods.^32,33^ Aspherical atom refinement can be performed using various quantum crystallographic methods, including Hirshfeld atom refinement (HAR).^34,35^ HAR uses aspherical atomic scattering factors obtained from the quantum-mechanical calculation of electron density (usually using DFT methods) to refine X-ray crystal structures^36,37^ and is the most advanced method of refining X-ray crystal structures, providing structural parameters for hydrogen atoms including both the H-atom positions and their anisotropic displacement parameters.^38,39^ Another advantage of HAR is the capability to include the crystal environment during the refinement, which can improve its quality when compared to IAM or MM/TAAM methods based on the pseudo-atom approach.^34^ Additionally, HAR seems to be effective even in the case of routine diffraction data.^40^

Despite that accurate and precise structural information may be obtained from HAR, there are only few studies dedicated to topics such as patterns of hydrogen bonds in the crystal, or the presence of structural clashes between the neighbouring atoms. Such information may allow to design of new drug formulations with improved bioavailability,^33^ since materials/drug properties are related to the electronic structure and accuracy of its determination. The previously mentioned papers focus on structural and biochemical studies on compounds with potential pharmaceutical properties including anticancer^41^ and antiviral activities.^42^ In one study dedicated to protein–ligand interactions, it was found that ED polarization of the ligand molecule in the protein binding pocket could be used to predict the electrostatic features of the binding itself. However, these experimental results are difficult to interpret.^43^ In other studies, application of HAR, or electron-density analysis, was rather limited since it was not the focus of the research. The protonation and its influence on hydrogen bonding in crystals of urea derivatives and their co-crystals was the subject of one study which utilised HAR among other methods.^44^

**Fig. 1:**
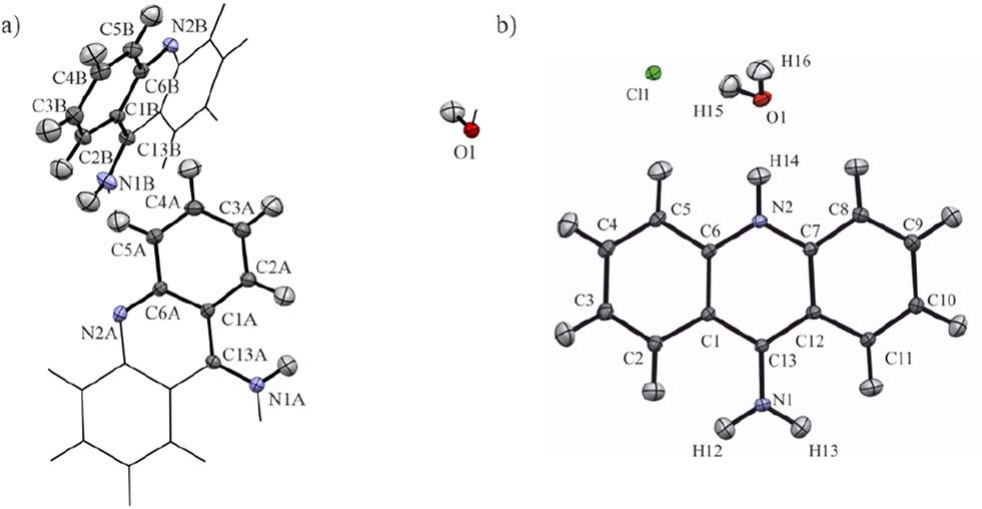
The asymmetric unit of (a) 9-aminoacridine hemihydrate (9aa*H_2_O) and (b) 9-aminoacridine hydrochloride monohydrate (9aa*HCl) with the labelling scheme. Atomic displacement ellipsoids are shown at a 50% probability level. The non-asymmetric unit of 9-aminoacridine hemihydrate is represented by sticks for the sake of clarity.

In this study, we decided to apply computational quantum chemistry tools, including periodic DFT calculations and topological analysis of electron density derived from molecular wavefunctions, to HAR to achieve better understanding the influence of protonation on electronic properties of 9-aminoacridine in the crystalline solid state. We investigated the influence of protonation on the intermolecular interactions and ED topological properties on the 9-aminoacridine moieties for 9-aminoacridine hemihydrate (9aa*H_2_O) and 9-aminoacridine hydrochloride monohydrate (9aa*HCl) (Fig. 1).

Protonation also modifies the intermolecular interactions by changing the proportion of the N⋯H and O⋯H contacts, and the number of C⋯H and H⋯H interactions. Theoretical calculations reveal that protonation stabilizes the crystal structure due to the presence of strong halogen and hydrogen bonds which compensates for the destabilizing effect of the interactions between two 9-aminoacridine moieties. Finally, we investigate the effect of 9aa protonation on its binding to selected DNA and protein molecules.

## 2. Experimental

### 2.1. Materials and crystallization procedures

**Fig. 2:**
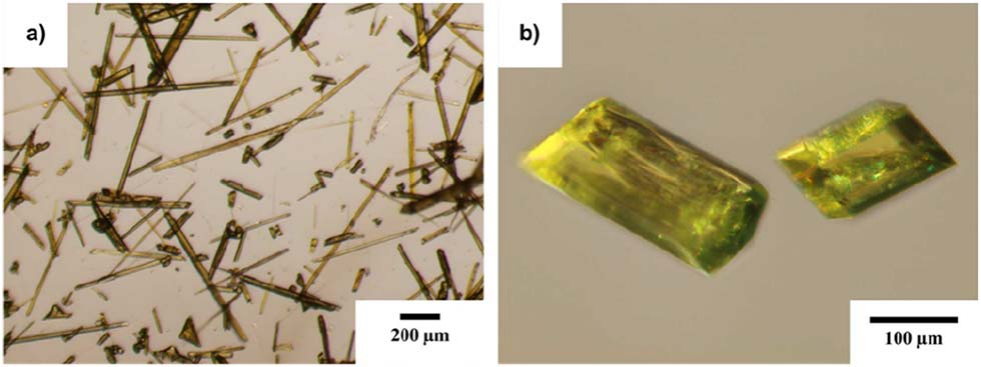
Overall view on the single crystals of 9-aminoacridine hemihydrate (a) and 9-aminoacridine hydrochloride monohydrate (b) grown from ethanol : water mixture (1 : 1; v : v).

The 9aa*H_2_O and 9aa*HCl were purchased from Sigma Aldrich and used without further purification. Small amount of both substances were dissolved in a mixture of ethanol and water (1 : 1; v : v) and then heated for several minutes. In the next step, the solutions were left to slowly evaporate at room temperature until the appearance of crystals. Typical representatives of single crystals from both samples are shown in Fig. 2.

### 2.2. Data collection

Data was collected for both crystals at different resolutions. For the 9aa*H_2_O dataset, we were only able to collect low-resolution data (Table 1) due to the weakly diffracting sample. X-ray data were taken at 100(2) K using an Agilent Technologies SuperNova Dual Source diffractometer (CuKα radiation, *λ* = 1.54184 Å). For the 9aa*HCl dataset, we have collected high-resolution X-ray diffraction data at 100(2) K on a Bruker AXS Kappa APEX II Ultra diffractometer equipped with a rotating anode (Mo Kα radiation, *λ* = 0.71073 Å). The data set was integrated with *CrysAlis* (CrysAlisPRO, Oxford Diffraction/Agilent Technologies UK Ltd, Yarnton, England) and *APEX2* (ref. 45) packages for the 9aa*H_2_O and 9aa*HCl datasets respectively. Data sets were also corrected for Lorentz and polarization effects.^46^ The absorption correction from crystal shape was applied to the 9aa*H_2_O and 9aa*HCl datasets respectively. Reflections were merged with *SORTAV*.^47^ Experimental details and refinement parameters for both compounds are summarized in Table 1.

**Table 1:** Experimental details of investigated compounds

	9aa*H_2_O	9aa*HCl
Crystal system	Tetragonal	Triclinic
Space group	*I*4_1_/*acd*	*P*1̄
Empirical formula	C_26_H_22_N_4_O_1_	C_13_H_13_ClN_2_O
Formula weight	406.48	248.70
*a*/Å	24.310(2)	7.7880(5)
*b*/Å	24.310(2)	8.7699(5)
*c*/Å	14.016(2)	9.7291(6)
*α*/°	90	111.057(2)
*β*/°	90	96.558(2)
*γ*/°	90	104.436(2)
Volume/Å^3^	8283.6(17)	585.08(6)
*Z*	16	2
*μ*/mm^−1^	0.647	0.310
*T*_max_	0.927	0.981
*T*_min_	0.888	0.958
*ρ*_calc_/mm^3^	1.304	1.412
*F*(000)	3424	260
Crystal size/mm^3^	0.08 × 0.09 × 0.14	0.18 × 0.20 × 0.31
*R*_int_	0.0384	0.0275
*R*_sigma_	0.0383	0.0259
*hkl* index ranges	27 : −29	16 : −16
	26 : −29	17 : −18
	10 : −16	20 : 0
Reflections collected	7758	11 578
2*θ* range for data collection	3.36 to 52.14	4.6 to 97.52
Temperature/K	100(2)	100(2)
X-ray wavelength/Å	1.54184	0.71073
Independent reflections	2017	11 578

### 2.3. Structure solution and refinement

The structural determination procedure for both crystal structures was carried out using *SHELX* package.^48–50^ The structures were solved with direct methods,^48^ and then successive least-squares refinements were performed based on full-matrix least-squares on *F*^2^ using *SHELXL*^49^ with the graphical interface of *Olex2* software.^51^ All H atoms were positioned geometrically with the C–H bond length equal to 0.93 Å for the aromatic hydrogen atoms, 0.85 Å for the hydroxyl hydrogen atom and 0.88 Å for the amine hydrogen atoms and constrained to ride on their parent atoms with *U*_iso_(H) = *xU*_eq_(C), where *x* = 1.2 for the aromatic and amine H atoms and *x* = 1.5 for the hydroxyl H atom.

**Fig. 3:**
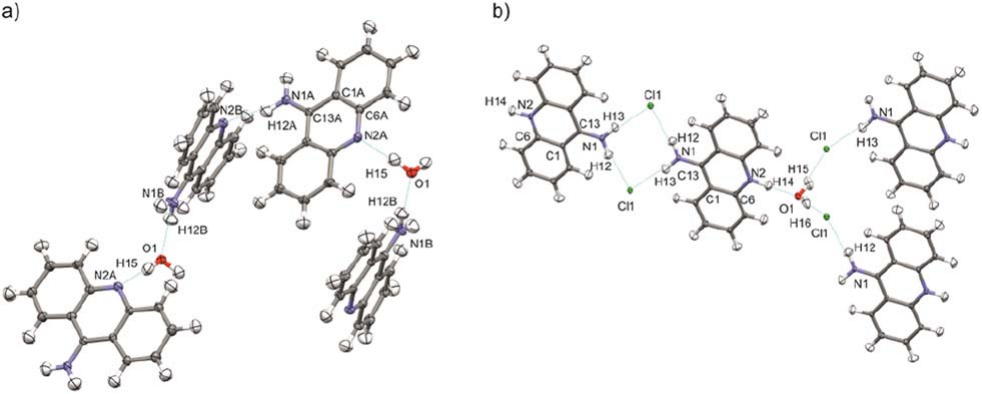
Selected clusters of molecules of (a) 9aa*H_2_O and (b) 9aa*HCl defined for HAR refinements with the atom labels for the investigated interactions.

Aspherical structural refinements (HAR approach) were carried out using *Discamb*,^52^ our in-house program, connected to the *Olex.refine* engine.^53^ The calculations of the molecular wavefunction were performed with the ORCA 5.0 package^54,55^ at the B3LYP^56^/cc-pVDZ^57^ levels of theory. The SCF calculations were performed for cluster of molecules, defined in such a way as to enable investigation of the intermolecular interactions present in the crystal structures as presented in Fig. 3. Also a cluster of charges and dipoles was applied during SCF calculations in order to simulate the crystal environment^58,59^ of all the neighbouring molecules, which have any atom within a radius of 16 Å from the central molecule. During refinement, all atomic positions were refined without any constraints or restraints applied. ADPs were computed and refined only for C, N and O, while H atoms were estimated using *SHADE3* server^60^ and fixed during the refinements.

Selected final statistics for all refinements are given in Table 1. More information can be found in the .cif files (deposition numbers 2270291–2270294†).

### 2.4. Topological analysis

The analysis of the electron density distribution obtained during HAR refinement was performed according to the Bader's Quantum Theory of Atoms in Molecules (QTAIM) formalism^61^ in *AIMAll* software.^62^

### 2.5. Theoretical calculations

Geometry optimization under periodic conditions was carried out with *Crystal09* (ref. 63) for the 9aa*H_2_O dataset and *Crystal17* (ref. 64) for the 9aa*HCl dataset, respectively, at the B3LYP^56^/cc-pVTZ^65^ level of theory with an employed Grimme dispersion^66,67^ and BSSE corrections.^68^ In both cases, we have used the lattice parameters and experimental geometry obtained from X-ray diffraction experiments and run optimization of the atomic positions only. Consequently, cohesive crystal lattice and dimer interaction energies based on those optimized geometries were calculated according to the case described for molecular crystals available at https://www.crystal.unito.it/website. Hirshfeld surface analysis was done with *CrystalExplorer17* (ref. 69) at the B3LYP/6-31G(d,p) level of theory for the geometry obtained from theoretical periodic calculations. Electrostatic potential (ESP)^70^ was computed using *Multiwfn*3.8 (ref. 71) program using a molecular wavefunction from the HAR refinement.

### 2.6. Macromolecular studies

Selected structures were downloaded from PDB database. To each structure hydrogen atoms were added, and local geometry optimisation was performed (heavy atoms in the protein or DNA molecules were fixed) using *Chimera 1.17* program and surface analyses for bound ligands were calculated in *CrystalExplorer17*. For DFT calculations selected fragments of structures were tailored in *ChimeraX*^72^ program and all computations were performed in *ORCA* 5.0 package using ChimeraX and the *SEQCROW*^73^ bundle as a graphical interface. Molecular wavefunction were calculated at the ωB97X-D3/def2-SVP^74,75^ level of theory using an implicit SMD solvent model.^76^ All ED functions were calculated in *Multiwfn* and visualised in *ChimeraX*.

## 3. Results and discussion

### 3.1. Crystal structures

The first deposition of crystal structures determined from single crystal XRD measurement at room temperature for both, the 9aa*H_2_O and 9aa*HCl, have been done by Chaudhuri in 1983 (ref. 77) and by Talacki in 1973,^78^ respectively. Here, we have reinvestigated the crystal structures of both forms at 100 K. Similarly, to the previously reported work, we have found that 9aa*H_2_O crystallized in the tetragonal, body centred *I*4_1_/*acd* space group with two halves of the 9aa molecules (hereafter called A and B molecules) and half of a water molecule in the asymmetric part of the unit cell (Fig. 1).

**Fig. 4:**
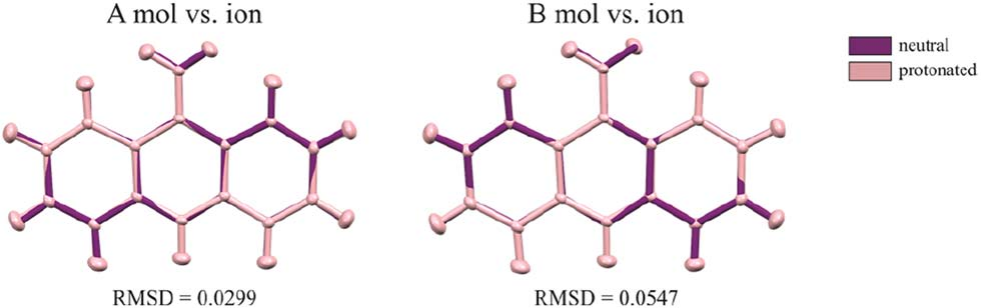
The conformational differences of the acridine visualized by superpositions of A and B neutral molecules *vs.* cation. RMSD values were calculated in mercury 2022.2.0 (build 353591).

On the other hand, 9aa*HCl crystallized in the triclinic *P*1̄ space group with one of the acridine and chloride ions and one water molecule in asymmetric part (Fig. 1). In both investigated systems, the anthracene ring is planar, with the amine group directed almost in its plane. Protonation virtually does not change acridine's geometry (Fig. 4), and the main differences between the acridine moiety for the neutral *vs.* protonated forms come from the slightly different orientations of the amine group. In the case of the 9aa*H_2_O, the dihedral angle between the planes defined by the anthracene ring and amine group is around 0.32° and 8.69° for A and B molecules, respectively. For the acridine cation this angle is equal to 8.35°, however, orientation of the amine group is different than those observed for the neutral form B molecule. Numerical values of the bond lengths can be found in the ESI (Tables S3 and S4†) or in the .cif files.

### 3.2. Comparison of different models of electron density

The details of the spherical and aspherical refinements are presented in Table 2. The advantage of using aspherical approaches over the IAM model^79^ is evident in both cases and supported by general refinement agreement indicators. However, applying aspherical refinement did not improve residual density values for the 9aa*H_2_O, contrary to the refinements of the 9aa*HCl. Differences in data resolution might cause this, and in the case of 9aa*H_2_O for which we collected only the low-angle data, the residual density is mainly located on the outermost parts of benzene rings. This residual electron density may suggest some structural disorder which is not resolvable here (Fig. S1†).

**Table 2:** Spherical and aspherical refinements details

	9aa*H_2_O		9aa*HCl	
	IAM	HAR	IAM	HAR
*R*(*F*^2^)	5.48%	4.30%	2.80%	1.86%
w*R*^2^	12.80%	9.10%	7.88%	4.26%
Data	2017	2017	11 578	11 578
Restraints	0	0	0	0
Parameters	153	177	206	193
GooF	1.041	0.911	1.064	1.178
Largest diff. peak/hole (e Å^−3^)	0.15/−0.29	0.21/−0.26	0.59/−0.33	0.36/−0.29

For the 9aa*HCl, the differences between residual density after IAM and HAR are more noticeable. According to the theoretical calculations, the residual density after aspherical refinement is mainly located on chloride anion and in the vicinity of oxygen lone pairs (Fig. S2†). Also, the X–H bond lengths are significantly improved after aspherical refinement with the bond lengths as those obtained by neutron diffraction studies (Tables S1 and S2†).

### 3.3. Electron density distribution

**Fig. 5:**
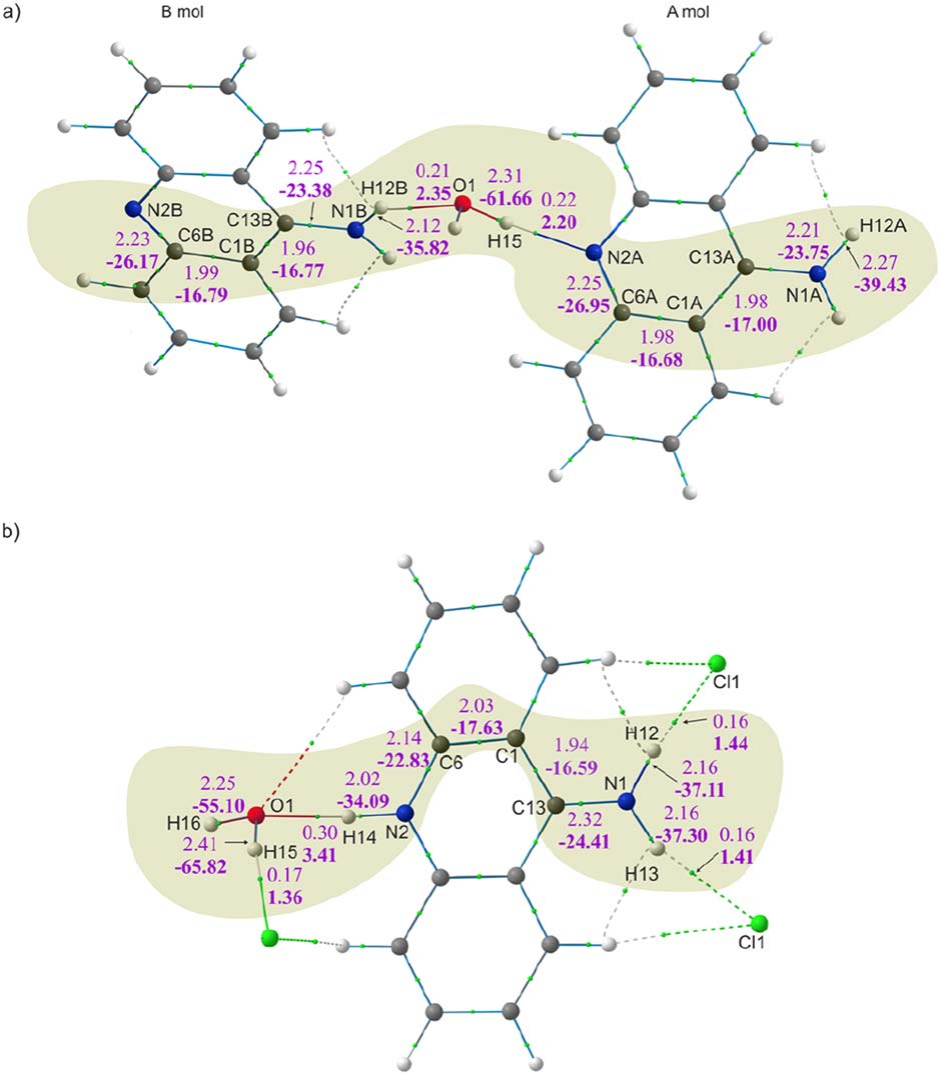
Molecular graphs after HAR with the electron density (upper) and its Laplacian (lower and bold) values at BCPs for the: (a) 9aa*H_2_O and (b) 9aa*HCl forms. BCPs are shown as small green spheres.

The values of the electron density (ED) and its Laplacian at the selected BCPs for the 9aa*H_2_O and 9aa*HCl are shown in Fig. 5 and Table 3. The bonding situation around the amino groups, water molecules and nitrogen atoms is different in neutral and protonated forms of 9aa, and we compare differences between these two systems. Firstly, there are no changes in the corresponding bond lengths due to the protonation effect within a particular type of refinement (Tables 3, S1 and S2†).

**Table 3:** Selected QTAIM parameters at BCPs for 9aa*H_2_O and 9aa*HCl

9aa*H_2_O					9aa*HCl				
Bond	*d* (Å)	*ρ*_BCP_ (e Å^−3^)	∇^2^*ρ*_BCP_ (e Å^−5^)	*ε*	Bond	*d* (Å)	*ρ*_BCP_ (e Å^−3^)	∇^2^*ρ*_BCP_ (e Å^−5^)	*ε*
N1A–H12A	1.01	2.27	−39.43	0.04	N1–H13	1.02	2.16	−37.30	0.03
N1A–C13A	1.34	2.21	−23.75	0.05	N1–H12	1.02	2.16	−37.11	0.03
C1A–C13A	1.42	1.98	−17.00	0.17	N1–C13	1.32	2.32	−24.41	0.07
C1A–C6A	1.42	1.98	−16.68	0.18	C1–C13	1.44	1.94	−16.59	0.15
C6A–N2A	1.35	2.25	−26.95	0.09	C1–C6	1.41	2.03	−17.63	0.20
N1B–H12B	1.03	2.12	−35.82	0.04	C6–N2	1.36	2.14	−22.83	0.07
N1B–C13B	1.34	2.25	−23.38	0.06	N2–H14	1.05	2.02	−34.09	0.03
C1B–C13B	1.43	1.96	−16.77	0.17	O1–H15	0.97	2.25	−55.10	0.02
C1B–C6B	1.42	1.99	−16.79	0.18	O1–H16	0.95	2.41	−65.82	0.02
C6B–N2B	1.36	2.23	−26.17	0.10					
O1–H15	0.96	2.31	−61.66	0.20					

Fig. 5 and Table 3 both show that the protonation of the nitrogen atom (N2A/B) do not have noticeable impact on the values of the ED at the BCP for N2A/B–C6A/B *vs.* N2–C6 bonds. The difference in electron density observed at this BCP after protonation is around 0.11 e Å^−3^ with very small differences visible in the Laplacian values (*ca.* 4 e Å^−5^). Further examination of the C1–C6 and C1–C13 bonds (see Table 3 and Fig. 5 for details) shows no significant changes in the values of the ED and its Laplacian after protonation.

When looking at the BCP values for the anthracene rings (Tables S3 and S4†), we can immediately see that the delocalization of the electron density in this region of the moiety is not observed. Instead, increases and decreases of charge distribution with a simultaneous slight change of the C–C bond lengths are observed. This pattern is preserved in both 9aa forms, despite the different molecular architectures of these crystal, and thus, indicating that even disparate patterns of intermolecular interactions have the same impact on ED distribution within the polycyclic system (Fig. S3†). Notably, the absence of pure delocalization of the electron density followed by a specific change of C–C bond lengths structures agrees with the previously published results for structures containing 9aa or its derivatives.

Furthermore, it appears that the primary differences in electron density distributions between 9aa*H_2_O and 9aa*HCl are in the amine group region and stemmed from the presence of the N–H⋯Cl interaction. These interactions cause an increase in the ED and its Laplacian at the BCP for the C13–N1 bond and decrease for the N1–H12/13 bond, when comparing the neutral A molecule and protonated forms (as shown in Fig. 5 and Table 3). This observation is slightly different when looking at the neutral B molecule since for both bonds the increase of ED at BCP is observed. For the water molecule, when examining the values at the BCP after protonation, a decrease in the ED and its Laplacian is observed only for one O–H bond, namely O1–H15 (Fig. 5 and Table 3). This may suggest that HAR is sensitive for this type of interactions, particularly because one would expect a non-equivalent behaviour of these two bonds due to different interactions with other atoms in their close vicinity (Fig. S4†).

The integration of the electron density over atomic basins also provided the charge and basin volumes of atoms (Table 4). In principle, protonation should change the curvature of regions bounded by zero-flux surfaces, and thus affects atomic charges basin volumes. As it is summarised in Table 4, the protonation of the N2A atom resulted in a change of its atomic charge and basin volume by −0.10 *e* and 2.58 Å^3^, respectively. However, when looking at other atoms, the effect of protonation appears to be small.

**Table 4:** Atomic charges *Q*_AIM_ and atomic basin volumes *V*_AIM_ for investigated compounds obtained from integration of the electron density over atomic basins

9aa*H_2_O			9aa*HCl		
Atom	*Q*_AIM_ (*e*)	*V*_AIM_ (Å^3^)	Atom	*Q*_AIM_ (*e*)	*V*_AIM_ (Å^3^)
N1A	−1.35	16.76	N1	−1.36	16.51
N2A	−1.23	15.56	N2	−1.33	13.98
H12A	0.51	2.53	H12	0.52	2.62
C13A	0.54	8.53	H13	0.52	2.67
N1B	−1.36	16.61	C13	0.62	8.30
N2B	−1.22	17.41	H14	0.57	2.16
H12B	0.50	3.71	O1	−1.34	19.71
C13B	0.56	8.50	H15	0.64	2.17
O1	−1.32	19.79	H16	0.65	2.06
H15	0.66	1.81	Cl1	−0.85	39.40

### 3.4. Intermolecular interactions

**Fig. 6:**
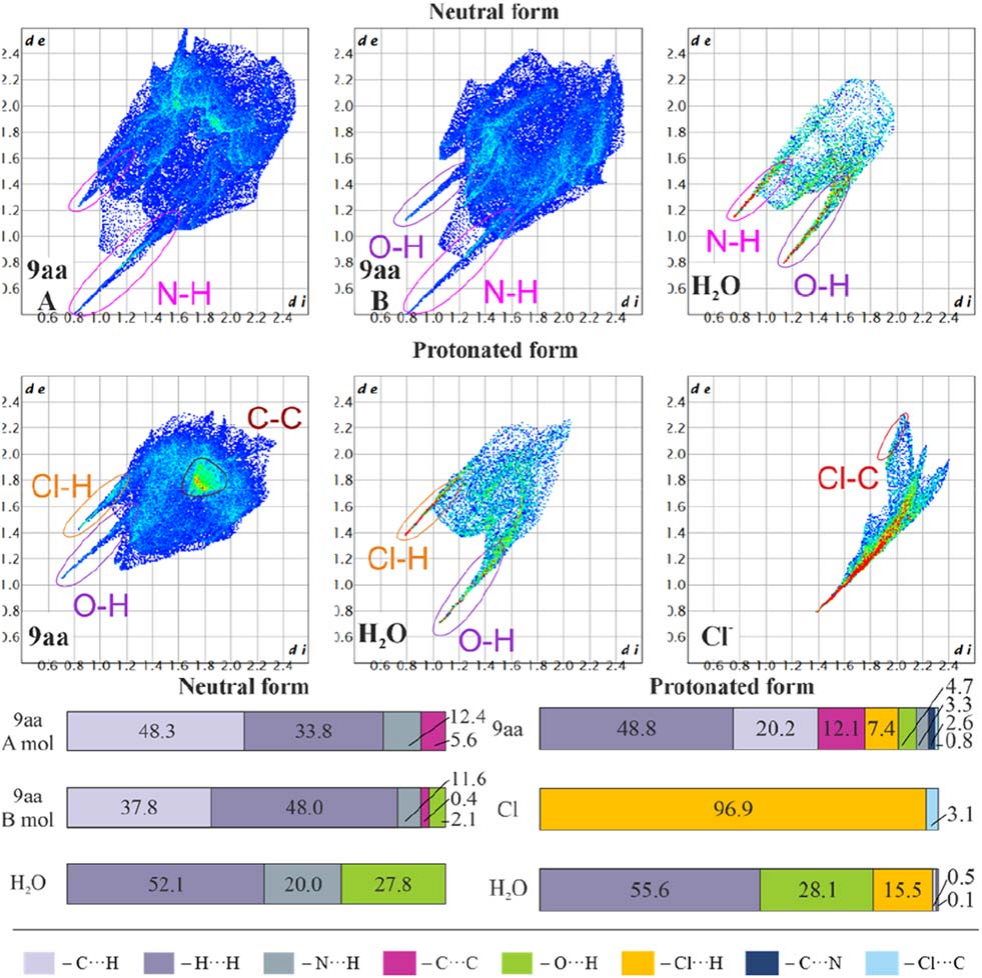
Hirshfeld surfaces: two-dimensional fingerprint plots (upper) and percentage contributions to the Hirshfeld surface area of each type of intermolecular contact (lower) for 9aa*H_2_O and 9aa*HCl.

Fig. 6 shows fingerprint plots and percentage contributions to the Hirshfeld surface of each type of intermolecular contact. One of the most prominent features in all the presented fingerprint plots is the presence of sharp “spikes” in their bottom regions. They are associated with the appearance of some strong O–H⋯Cl, N–H⋯O and O–H⋯N hydrogen bonds in the crystal structures. The positions and shapes of the spikes before and after protonation are different for individual moieties, indicating that protonation and presence of the chloride anion affect hydrogen bond patterns, which is consistent with previously reported results.^80^

In the case of the acridine moiety, the introduction of an H atom has a significant effect on the contribution of N⋯H and O⋯H contacts. Due to the different molecular arrangement of the 9aa moiety in both forms, 9aa participates in fewer N⋯H contacts after protonation (12.4/11.6% *vs.* 3.3% for the 9aa*H_2_O mol A/B and 9aa*HCl, respectively, Fig. 6). This difference is also reflected in the two-dimensional fingerprint plots, where the sharp spike associated with N⋯H contacts disappears in the 9aa*HCl. On the contrary, the contribution of the O⋯H contacts almost doubled with the protonation (2.1% *vs.* 4.7% for the 9aa*H_2_O (mol B) and 9aa*HCl, respectively, as shown in Fig. 6).

Another visible effect of protonation for the 9aa moiety is a reduced contribution of the C⋯H contacts from 48.3/37.8% to 20.2% (Fig. 6) in favour of an increased contribution of the H⋯H contacts (33.8/48.0% *vs.* 48.8% for the 9aa*H_2_O (mol A/B) and 9aa*HCl, respectively, Fig. 6). Additionally, the area of the two-dimensional fingerprint plot associated with the C⋯C contacts looks different after protonation (Fig. 6). The density of the plot in this area is substantially higher which is evidence of larger fraction of surface points participating in C⋯C contacts (π-stacking) in the protonated form than in the neutral one. Naturally, for the 9aa*HCl, contributions of the Cl⋯H, Cl⋯C, and C⋯N contacts are observed, mostly due to the presence of a chloride anion interacting with the –NH_2_ group.

The influence of protonation on the acridine molecule is also visible in the contribution of interatomic contacts for water molecules, which is reflected in the increased contribution of the H⋯H contacts (52.1% *vs.* 55.6% for the 9aa*H_2_O and 9aa*HCl, respectively, Fig. 6). The change in the contribution of the H⋯H contacts is small when looking at merely its numerical values, but a closer examination of two-dimensional fingerprint plots suggests a more densely packed arrangement of water molecules before protonation. On the other hand, protonation also results in slightly closer-packed planes of the 9aa moieties in crystal structures (Fig. S5†), which is also reflected in the two-dimensional fingerprint plots (the sum of the *d*_e_ and *d*_i_ for the protonated form is smaller than for the neutral form, Fig. 6). In summary, the overall crystal packing seems to be less crowded for 9aa*HCl, than for 9aa*H_2_O (Fig. S5†).

The total crystal lattice cohesive energies are −116.71 and −241.87 kJ mol^−1^ for the 9aa*H_2_O and 9aa*HCl, respectively. The energy decreases significantly after protonation, indicating its stabilising character. The tighter packing of the layers defined by the acridine ions for the 9aa*HCl form results in the formation of higher number of the weak intermolecular interactions between 9aa moieties than for the 9aa*H_2_O form, which is illustrated with more H⋯H contacts than C⋯H or N⋯H ones after protonation (as shown in Fig. 6).

**Fig. 7:**
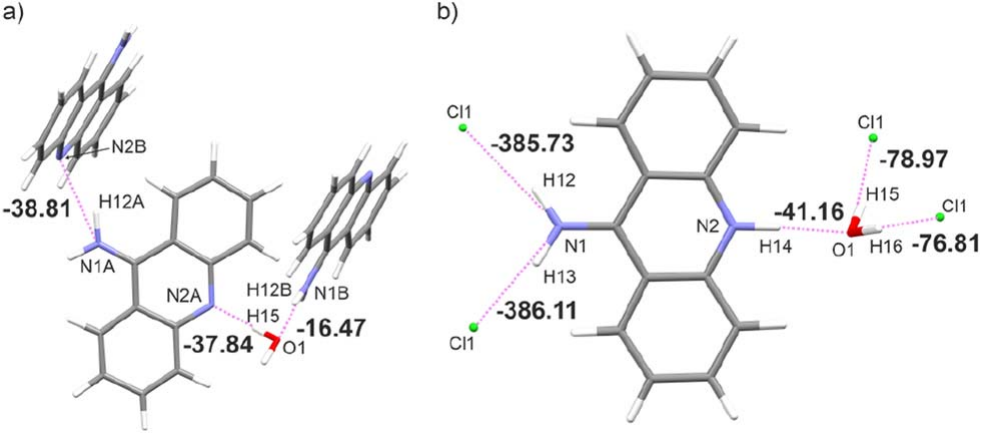
Dimer energies in kJ mol^−1^ of interacting molecules for: (a) 9aa*H_2_O and (b) 9aa*H_2_O.

This generally contributes to the overall destabilisation of the crystal lattice, with dimers defined between 9aa cations having energies of 166.76 kJ mol^−1^ and 145.69 kJ mol^−1^ (Fig. S6†). In this case, the most stabilising interactions in the crystal lattice are strong N–H⋯Cl, N–H⋯O and O–H⋯Cl hydrogen bonds (Fig. 7b). The chloride anion appears to act as a “molecular glue” that holds the entire crystal lattice together, with energy values of approximately −386.0 kJ mol^−1^ and −78.0 kJ mol^−1^, mostly due to electrostatically-assisted N–H⋯Cl and O–H⋯Cl contacts, respectively. The interaction energy between 9aa cation and a water molecule has a typical value of −41.16 kJ mol^−1^, mostly associated with the strong N–H⋯O hydrogen bond. Furthermore, the less crowded molecular arrangement observed in 9aa*HCl may reduce the number of repulsive interactions in the crystal lattice, increasing its stabilising character. In contrast, the crystal structure of the 9aa*H_2_O is mainly stabilised *via* weak C–H⋯π contacts observed between the 9aa molecules.

The values of such interaction energies range from −6.48 kJ mol^−1^ to −16.67 kJ mol^−1^ (Fig. S7†) and the largest interaction energies were found between the two 9aa molecules and 9aa A and water moiety, mostly due to the N–H⋯N and the O–H⋯N H-bonds, respectively (Fig. 7a). Nonetheless, these interactions are not the dominant contributors to the Hirshfeld surface, and the water molecule interacts with two acridine molecules with different strengths (Fig. 7a).

To achieve a more elaborate description of hydrogen bonding in the studied systems, we have also examined topological properties of the electron density at BCP and the source contribution^81^ to the BCP (Table 5) for all hydrogen bonds. The source function,^81^ which is highly sensitive to electron density perturbations, can reveal subtle changes in local electron density, and thus be a valuable tool for characterising bonding features of hydrogen bonds.^82,83^

Topological properties at the critical points and source contributions to the critical point of hydrogen bonds for the 9aa*H_2_O and 9aa*HCl

**Table:** 

9aa*H_2_O						
**Hydrogen bonds**	**D⋯A (Å)**	**H⋯A (Å)**	**DHA (°)**	***ρ***_**BCP**_ **(e Å^−^** ^**3**^ **)**	**∇** ^**2**^ ***ρ***_**BCP**_ **(e Å^−^** ^**5**^ **)**	***H***_**r**_ **(Haa**_**0**_ **^−^** ^**3**^ **)**
O1–H15⋯N2A	2.869	1.909	174.8	0.22	2.20	0.00
N1B–H12B⋯O1	2.828	1.895	148.7	0.21	2.35	0.00
N1A–H12A⋯N2B	3.043	2.064	163.9	0.16	1.52	0.00

**Hydrogen bonds**	***S*(D) (%)**	***S*(H) (%)**	***S*(A) (%)**	***S*(D + H + A) (%)**		
O1–H15⋯N2A	61.04	−22.01	5.58	44.61		
N1B–H12B⋯O1	54.54	−24.71	44.80	74.63		
N1A–H12A⋯N2B	49.80	−34.91	−10.83	4.06

**Table:** 

9aa*HCl						
**Hydrogen bonds**	**D⋯A (Å)**	**H⋯A (Å)**	**DHA (°)**	***ρ***_**BCP**_ **(e Å^−^** ^**3**^ **)**	**∇** ^**2**^ ***ρ***_**BCP**_ **(e Å** ^**−5**^ **)**	***H***_**r**_ **(Haa**_**0**_ **^−^** ^**3**^ **)**
O1–H16⋯Cl1	3.152	2.212	176.6	0.17	1.63	0.00
O1–H15⋯Cl1	3.159	2.192	169.12	0.16	1.60	0.00
N1–H13⋯Cl1	3.22	2.264	155.43	0.16	1.41	0.00
N1–H12⋯Cl1	3.217	2.249	157.35	0.16	1.44	0.00
N2–H14⋯O1	2.765	1.718	174.96	0.30	3.41	0.00

**Hydrogen bonds**	***S*(D) (%)**	***S*(H) (%)**	***S*(A) (%)**	***S*(D + H + A) (%)**		
O1–H16⋯Cl1	64.41	−29.69	51.51	86.24		
O1–H15⋯Cl1	70.36	−35.68	51.77	86.45		
N1–H13⋯Cl1	43.95	−30.17	48.82	62.60		
N1–H12⋯Cl1	43.48	−28.96	49.08	63.60		
N2–H14⋯O1	27.13	−3.15	38.19	62.17		

The overview of the topological properties of hydrogen bonds is presented in Table 5, and all of them were identified as closed-shell interactions (small value of the electron density and small and positive value of the Laplacian of electron density) of relatively medium-strength (small value of the total energy density, *H*_r_).^84,85^ For the 9aa*H_2_O, the O1–H15⋯N2A hydrogen bond is the strongest one among the all-existing H-bonds in the crystal structure in terms of the H⋯A distance and topological properties. Similarly, in the case of the 9aa*HCl, the strongest hydrogen bond is the N2–H14⋯O1 interaction. Noteworthy, protonation does not change either the strength or the character of the hydrogen bonds.

The percentage contribution of the hydrogen atoms to the electron density at the bond critical points for the studied hydrogen bonds is negative in all cases (Table 5). This indicates the electrostatic character of these bonds, and is also typical for the polarization-assisted hydrogen bonds.^81^ In almost all cases, the source contribution from the atom triad *S*(D + H + A)% has its value above 50% (the exceptions are O1–H15⋯N2A and N1A–H12A⋯N2B interactions) confirming its medium strength character.^84^ The *S*(D)% is larger than *S*(A)% for all hydrogen bonds present in the 9aa*H_2_O. For 9aa*HCl this condition was fulfilled for the interactions between water and chloride ion (Table 5). The last two criteria are rather characteristic for the isolated hydrogen bonds. In general, these interactions can be classified as isolated hydrogen bonds with some features of polarization-assisted hydrogen bonds supported by electrostatic forces. However, the polarization assistance is larger for 9aa*HCl, especially for the N1–H13⋯Cl1, N1–H12⋯Cl1 and N1–H14⋯O1 hydrogen bonds, where *S*%(D) is smaller than *S*%(A) contribution.

### 3.5. Intermolecular interactions in macromolecular systems

Since some acridines and their derivatives display biological activities and interact with certain macromolecules such as double-stranded DNA^86^ or proteins,^87^ we decided to include a short analysis of the 9aa interatomic interactions in existing macromolecular complexes to check if any significant changes could occur upon protonation. Due to lower resolution and accuracy of available X-ray structures of proteins, a reasonable approach to study intermolecular interactions with 9aa in a quantitative manner is to use Hirshfeld surfaces. We selected two entries from PDB database, namely 3tzb (quinone oxidoreductase)^88^ and 6o4x (human acetylcholinesterase)^89^ for further studies (Fig. S8 and Table S5†). Similarly, to previously discussed crystal structures, one of the leading features is the presence of sharp spikes in the bottom parts of 2D fingerprint plots, which are associated with the appearance of strong and medium O⋯H and C–H⋯O hydrogen bonds. Various π-stacking interactions also are present including T-shaped and sandwich-forms as well as cationic stacking. The two main changes in intermolecular interactions were observed. (i) Protonated 9aa in acetylcholinesterase lead to formation of the additional H-bond between N10 and the carbonyl group in the peptide bond of a histidine residue. (ii) Protonation of 9aa changes the character of stacking interactions between the NH_2_/NH_3_^+^ group of 9aa and a Trp residue, from T-shaped (neutral) to cationic (charged) in the quinone oxidoreductase.

**Fig. 8:**
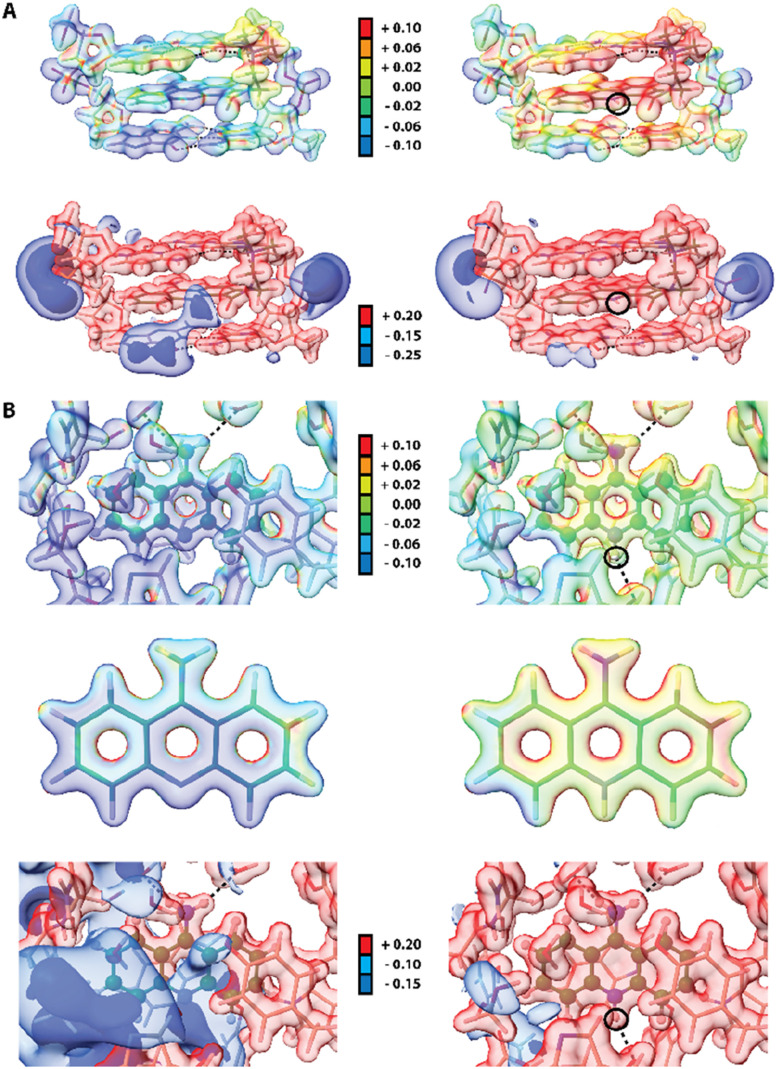
Effect of protonation on electrostatic properties of macromolecular complexes of 9aa and 9AD. In each case the electrostatic potential (ESP) is mapped on isosurfaces of electron density (contour 0.08 e A^−3^) prepared using a rainbow gradient colouring scheme where the lowest ESP values are blue and the highest are red (−0.1 to +0.1 *E*_h_ e^−1^). Isosurfaces of electrostatic potential at several selected values are also presented. H-bonds are depicted as black dotted lines and black circles indicate a ligand protonation site. (A) Complex of DNA and 9AD in its neutral (left) and protonated (right) form. ESP mapped onto electron density (top) and isosurfaces of ESP (bottom). (B) 9aa bound to human acetylcholinesterase in its neutral (left) and protonated (right) form (top). ESP mapped onto electron density for the ligand binding site (top) and 9aa extracted from the complex. (Middle) Isosurfaces of ESP are presented at the bottom. 9aa is depicted in ball and stick representation.

Since electrostatic potential (ESP) often plays a vital role in molecular recognition in biological systems^90,91^ we also compute ESP for two macromolecular complexes: previously mentioned human acetylcholinesterase and DNA complexed by 9-amino-*N*-(2-dimethylaminoethyl)acridine-4-carboxamide (9AD, PDB entry 465d).^92^ The results indicate significant change in electrostatic potential upon protonation of the ligands (Fig. 8). Since 9AD intercalates between DNA pairs changes in protonation state of its aromatic system led to a visible impact on the charge distribution in the DNA molecule, while the electron density itself is virtually unchanged except for the very close proximity of the protonation site. In physiological condition 9AD exists almost exclusively in the protonated form, which has a stronger affinity to the negatively charged DNA than the neutral form. A similar situation occurs in acetylcholinesterase where protonation of 9aa modifies the charge distribution in the entire binding site. Contrastingly, due to presence of large and negatively charged chlorine anions in the crystal structure of protonated 9aa, the changes in ESP are much less noticeable upon protonation of 9aa in the crystal state (Fig. S9†). For both complexes protonation does not have a significant effect on ED Laplacian and Electron Localization Function (ELF) beyond the ligand molecule itself. However aromatic residues near the 9aa molecule are affected by protonation to some extend which is reflected in Laplacian changes in the aromatic residues in the ligand vicinity.

## 4. Conclusions

We have analysed the influence of the protonation of the 9-aminoacridine moiety in terms of quantitative and qualitative investigations of intermolecular interactions present in the studied crystal structures as well as topological properties obtained from the theoretical electron densities.

Our investigation was focused on the regions of the N-atom and amine group of 9-aminoacridine. Detailed examination of the electron density distribution and bond lengths due to protonation showed that protonation barely changes the electron distribution around protonated N-atoms. Also, no significant changes in the corresponding bond lengths were observed.

However, the ED distribution in the proximity of the amine group and water molecule is changed, and a decrease of the ED at BCP for N/O–H bonds is visible, which is related to the presence of the chloride anion. In contrast, our examination of the atomic charges showed that protonation influences only the atomic charge of the protonated N2 atom. Investigation of the electron density at BCP for hydrogen bonds revealed their medium-strength character, however, we have obtained an unconventional set of source function contributions from the donor, acceptor, and the hydrogen atoms. Based on this analysis, we can conclude that investigated hydrogen bonds are isolated, with some polarization features and contribution of electrostatic forces, which increases its polarization effect for the protonated form.

Protonation changes the molecular architecture of the crystal structures, and thus modifies the intermolecular interactions. The analysis of the Hirshfeld surface and 2D fingerprints plots revealed that the different orientation of the acridine moiety after protonation has a significant effect on the proportion of N⋯H and O⋯H contacts. Namely, 9aa participates in fewer N⋯H contacts after protonation. On the contrary, the contribution of the O⋯H contacts almost doubled upon protonation. In addition, protonation also changed the number of weak interactions: the number of C⋯H interactions decreased while the number of H⋯H interactions increased.

Theoretical calculations of energetic features for dimers and crystal lattice proved the differences between neutral and protonated forms of this compound. Protonation stabilizes crystal structure due to the presence of strong hydrogen bonds, which compensate destabilizing effect associated with the interactions between two 9-aminoacridine cations. In general, in the case of studied systems, electrostatic forces have a dominant role in the stabilization of crystal lattice. Additionally, crystal structure after protonation displays less molecular crowding, decreasing the repulsive forces.

The results of our work agree with the previously reported differences observed between the neutral and protonated forms for certain APIs.^23,24,87^ Each form of 9aa should have different solubility in physiological conditions. Due to the presence of strong repulsive forces in the crystal lattice of protonated 9aa, it should have greater solubility in polar solvents and be more likely to host more water molecules. Quantum crystallography can be successfully used in investigation of charge distribution and may lead to improvement in drug design and help in the prediction of crystal structure properties.

## Author contributions

SP: crystallization of 9aa neutral, XRD experiment for 9aa neutral, data reduction for the 9aa neutral, HARs for 9aa neutral and protonated, QTAIM/SF analyses for all refinements, calculations in CE, Crystal09 and Crystal17, conceptualization and writing original draft, preparation of all tables and figures presented in publication and ESI.† MZ: writing of the introduction, text reviewing, macromolecular studies AM: XRD experiment for 9aa protonated, data reduction for 9aa protonated DT: crystallization of 9aa neutral, assistance in XRD experiments, conceptualization KW: funding, supervising, editing, correcting of the text, discussion of results.

## Conflicts of interest

There are no conflicts of interest to declare.

## Supplementary Material

RA-014-D3RA08081A-s001

RA-014-D3RA08081A-s002
