# Supplementary material for: Influence of N-protonation on electronic properties of acridine derivatives by quantum crystallography
Source: RSC Adv. 2024 Feb 12;14(8):5340–50. doi: 10.1039/d3ra08081a (PMC10859733; doi:10.1039/d3ra08081a)
Supplement: RA-014-D3RA08081A-s001 [file RA-014-D3RA08081A-s001.pdf]

Electronic Supplementary Material (ESI) for RSC Advances.
This journal is © The Royal Society of Chemistry 2024

Influence of N-protonation on electronic properties of acridine derivatives by quantum crystallography

Sylwia Pawlędzio[1,2*], Marcin Ziemniak[2], Damian Trzybiński[2], Mihails Arhangelskis[2], Anna Makal[2], Krzysztof Woźniak[2*]

Corresponding author: pawledzios@ornl.gov, kwozniak@chem.uw.edu.pl

[1] Neutron Scattering Division, Oak Ridge National Laboratory, Oak Ridge, TN 37831, USA

[2] Biological and Chemical Research Centre, Department of Chemistry, University of Warsaw, Żwirki i Wigury 101, 02-093 Warszawa, Poland

Table S1. *X*–H bond lengths in Å after HAR and TAAM refinements for **9aa*H$_2$O** form.

Bond	IAM	HAR
N1A–H12A	0.86(2)	1.01(2)
C2A–H2A	0.93	1.091(19)
C3A–H3A	0.93	1.074(19)
C4A–H4A	0.93	1.124(19)
C5A–H5A	0.93	1.077(19)
H12B–N1B	0.89(2)	1.031(19)
C2B–H2B	0.93	1.075(17)
C3B–H3B	0.93	1.050(18)
C4B–H4B	0.93	1.121(19)
C5B–H5B	0.93	1.098(19)
O1–H15	0.85	0.96(2)

Table S2. *X*–H bond lengths in Å after HAR and MM refinements for **9aa*HCl** form.

Bond	IAM	HAR
O1–H16	0.790(13)	0.948(7)
O1–H15	0.849(12)	0.973(6)
N2–H14	0.886(11)	1.050(5)
N1–H13	0.880(10)	1.021(5)
N1–H12	0.879(10)	1.022(5)
C11–H11	0.933(10)	1.085(4)
C5–H5	0.953(10)	1.075(5)
C2–H2	0.958(10)	1.088(5)
C9–H9	0.964(10)	1.079(5)
C4–H4	0.952(9)	1.066(4)
C8–H8	0.986(10)	1.072(5)
C10–H10	0.999(9)	1.085(5)
C3–H3	0.937(10)	1.083(5)

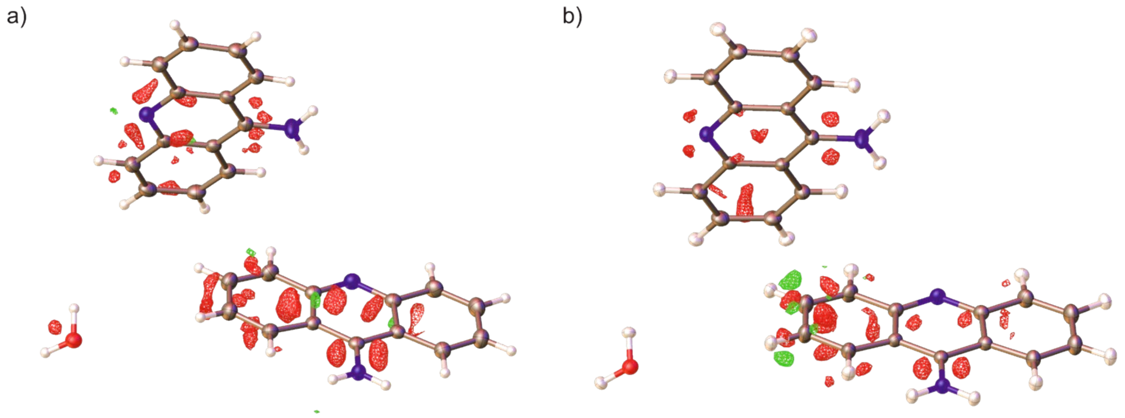

Figure S1. 2D visualization of the residual density (eÅ^{-3}) after a) IAM and b) HAR models for **9aa*H$_2$O** form.

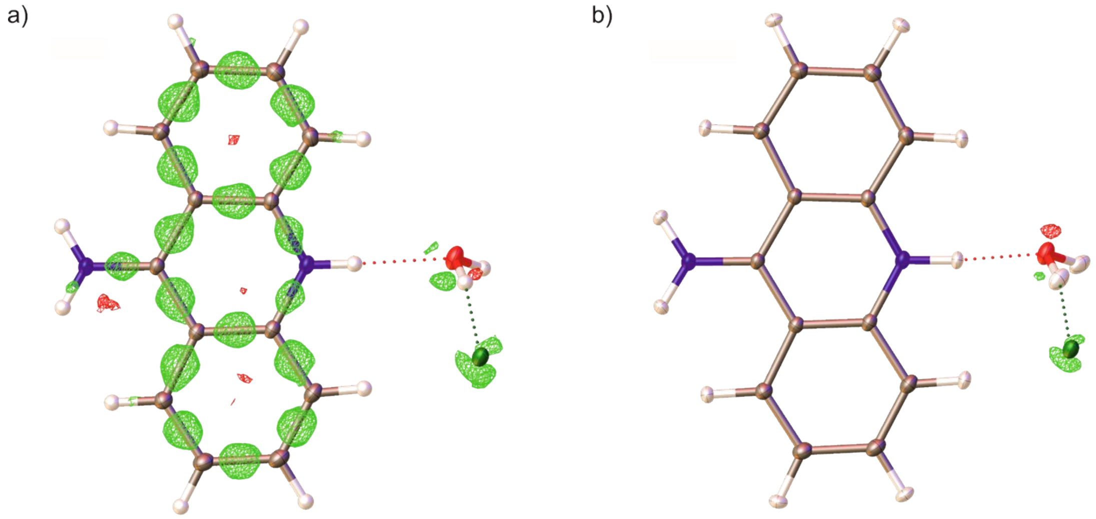

Figure S2. 2D visualization of the residual density (eÅ^{-3}) after a) IAM and b) HAR models for **9aa*HCl** form.

Table S3. QTAIM parameters at BCPs for **9aa*H_2O** from HAR model.

	HAR			
**Bond**	**d (Å)**	**ρ_{BCP} (eÅ^{-3})**	**$\nabla^2\rho_{BCP}$ (eÅ^{-5})**	**ε**
N1A–C13A	1.344(2)	2.21	-23.75	0.05
N1A–H12A	1.01(2)	2.27	-39.43	0.04
N2A–C6A	1.351(2)	2.25	-26.95	0.09
C1A–C2A	1.425(2)	1.95	-16.28	0.16
C2A–C3A	1.366(2)	2.17	-19.55	0.26
C3A–C4A	1.415(2)	2.00	-17.25	0.15
C4A–C5A	1.366(2)	2.18	-19.67	0.25
C5A–C6A	1.426(2)	1.98	-17.19	0.17
C1A–C6A	1.425(2)	1.98	-16.68	0.18
C1A–C13A	1.425(1)	1.98	-17.00	0.17
C2A–H2A	1.091(19)	1.89	-25.08	0.02
C3A–H3A	1.074(19)	1.91	-25.82	0.02
C4A–H4A	1.124(19)	1.76	-22.20	0.01
C5A–H5A	1.077(19)	1.96	-27.13	0.02
N1B–H12B	1.031(19)	2.12	-35.82	0.04
N1B–C13B	1.336(2)	2.25	-23.38	0.06
N2B–C6B	1.357(1)	2.23	-26.17	0.10
C1B–C2B	1.425(2)	1.95	-16.27	0.17
C2B–C3B	1.369(2)	2.16	-19.27	0.25
C3B–C4B	1.417(2)	1.99	-17.20	0.15
C4B–C5B	1.369(2)	2.16	-19.37	0.25
C5B–C6B	1.432(2)	1.96	-16.81	0.17
C1B–C6B	1.423(2)	1.99	-16.79	0.18
C1B–C13B	1.429(2)	1.96	-16.77	0.17
C2B–H2B	1.075(17)	1.95	-26.71	0.02
C3B–H3B	1.050(18)	2.02	-28.99	0.02
C4B–H4B	1.121(19)	1.77	-22.39	0.01
C5B–H5B	1.098(19)	1.87	-24.77	0.02
O1–H15	0.96(2)	2.35	-57.45	0.02

Table S4. QTAIM parameters at BCPs for **9aa*HCl** form HAR model.

		HAR		
**Bond**	**d (Å)**	**ρ_{BCP} ($eÅ^{-3}$)**	**$\nabla^2\rho_{BCP}$ ($eÅ^{-5}$)**	**ε**
N2–C6	1.3607(2)	2.14	-22.74	0.07
N2–C7	1.3605(2)	2.14	-22.83	0.07
N2–H14	1.050(5)	2.02	-34.09	0.03
N1–C13	1.3233(2)	2.32	-24.41	0.07
N1–H13	1.021(5)	2.16	-37.11	0.03
N1–H12	1.022(5)	2.16	-37.30	0.03
C12–C7	1.4110(2)	2.03	-17.63	0.20
C1–C6	1.4098(2)	2.03	-17.52	0.20
C1–C2	1.4187(2)	1.97	-16.52	0.17
C2–C3	1.3733(2)	2.15	-19.12	0.24
C3–C4	1.4105(3)	2.01	-17.39	0.16
C4–C5	1.3742(3)	2.15	-19.14	0.24
C5–C6	1.4149(2)	2.01	-17.45	0.18
C7–C8	1.4153(2)	2.01	-17.52	0.18
C8–C9	1.3729(3)	2.14	-19.03	0.24
C9–C10	1.4120(3)	2.02	-17.48	0.16
C10–C11	1.3730(2)	2.14	-19.10	0.24
C11–C12	1.4201(2)	1.97	-16.61	0.17
C12–C13	1.4370(2)	1.94	-16.59	0.15
C1–C13	1.4380(2)	1.94	-16.63	0.15
C2–H2	1.088(5)	1.92	-26.56	0.01
C3–H3	1.083(5)	1.89	-25.08	0.02
C4–H4	1.066(4)	1.93	-26.54	0.01
C5–H5	1.075(5)	1.95	-27.21	0.02
C8–H8	1.072(5)	1.94	-26.97	0.02
C9–H9	1.079(5)	1.98	-27.79	0.01
C10–H10	1.085(5)	1.90	-25.83	0.02
C11–H11	1.085(4)	1.91	-26.25	0.01
O1–H16	0.948(7)	2.25	-55.10	0.02
O1–H15	0.973(6)	2.41	-65.82	0.02

Table S5. Contribution of each type of interatomic contact to the overall Hirshfeld surface for aa9 residues (either in neutral form or protonated) in selected PDB entries expressed in percent [%].

**Type of contact**	**6o4x (neutral)**	**6o4x (protonated)**	**3tzb (neutral)**	**3tzb (protonated)**
N···H	4.9	3.2	4.9	4.7
C···H	18.4	18.1	17.7	18.2
O···H	12.6	12.9	16.8	16.7
H···H	49.0	51.5	49.6	50.6
C···N	2.1	1.9	2.7	1.9
C···O	2.4	2.2	0.7	0.7
C···C	9.0	8.6	6.9	6.6
N···O	1.5	1.5	0.0	0.0
N···N	0.0	0.0	0.6	0.5

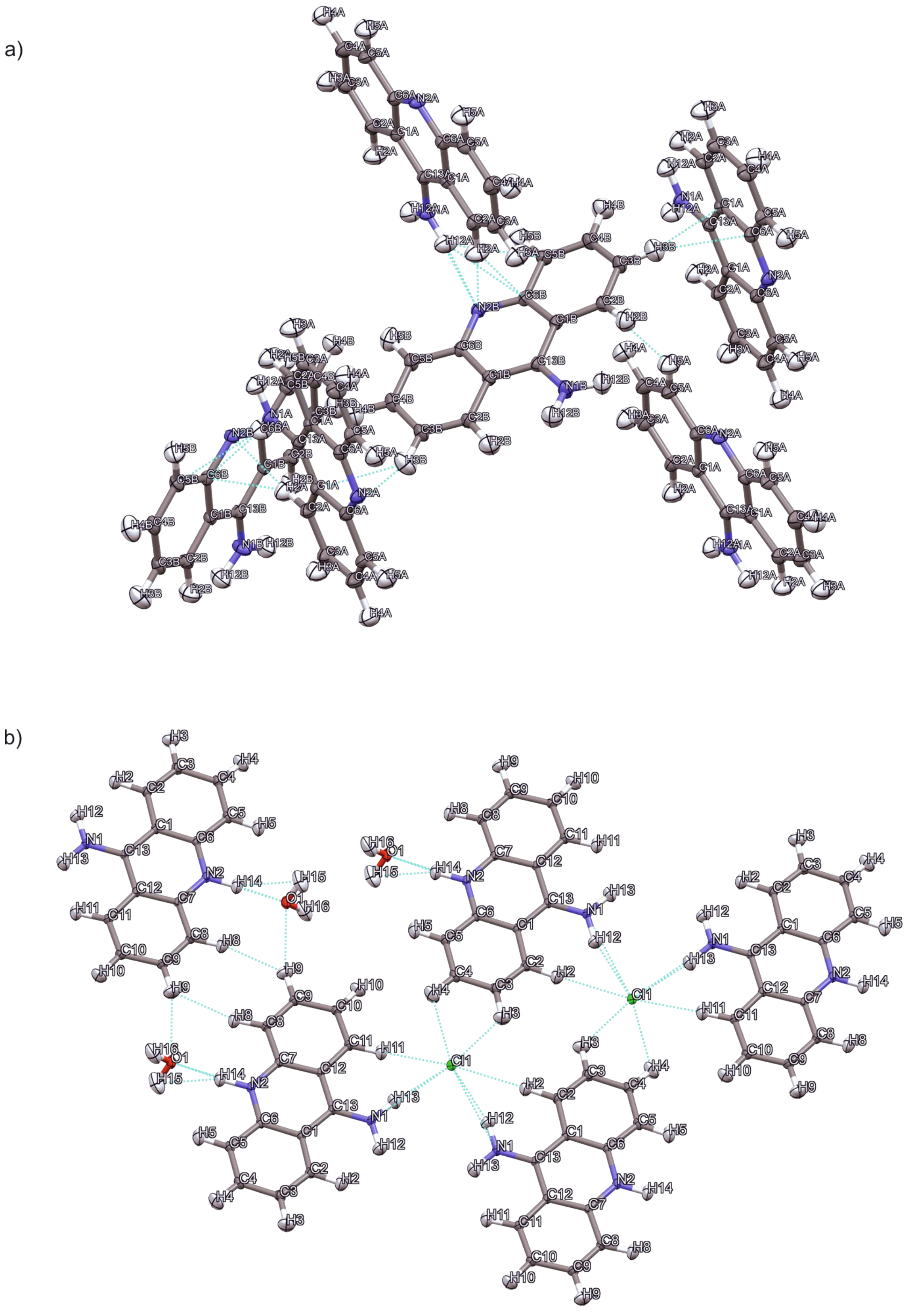

Figure S3. Molecular arrangements of a) **9aa*H_2O** and b) **9aa*HCl** forms showing the intermolecular interactions of aminacrine.

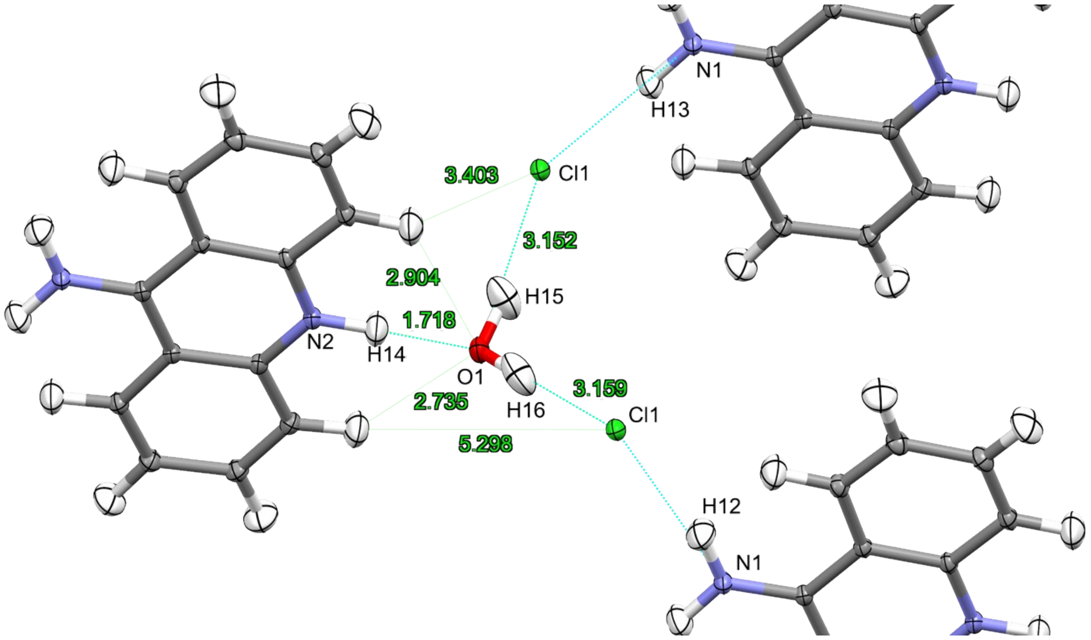

Figure S4. Distances of intermolecular interactions for a water molecule in the crystal structure of **9aa*HCl**.

a)

b)

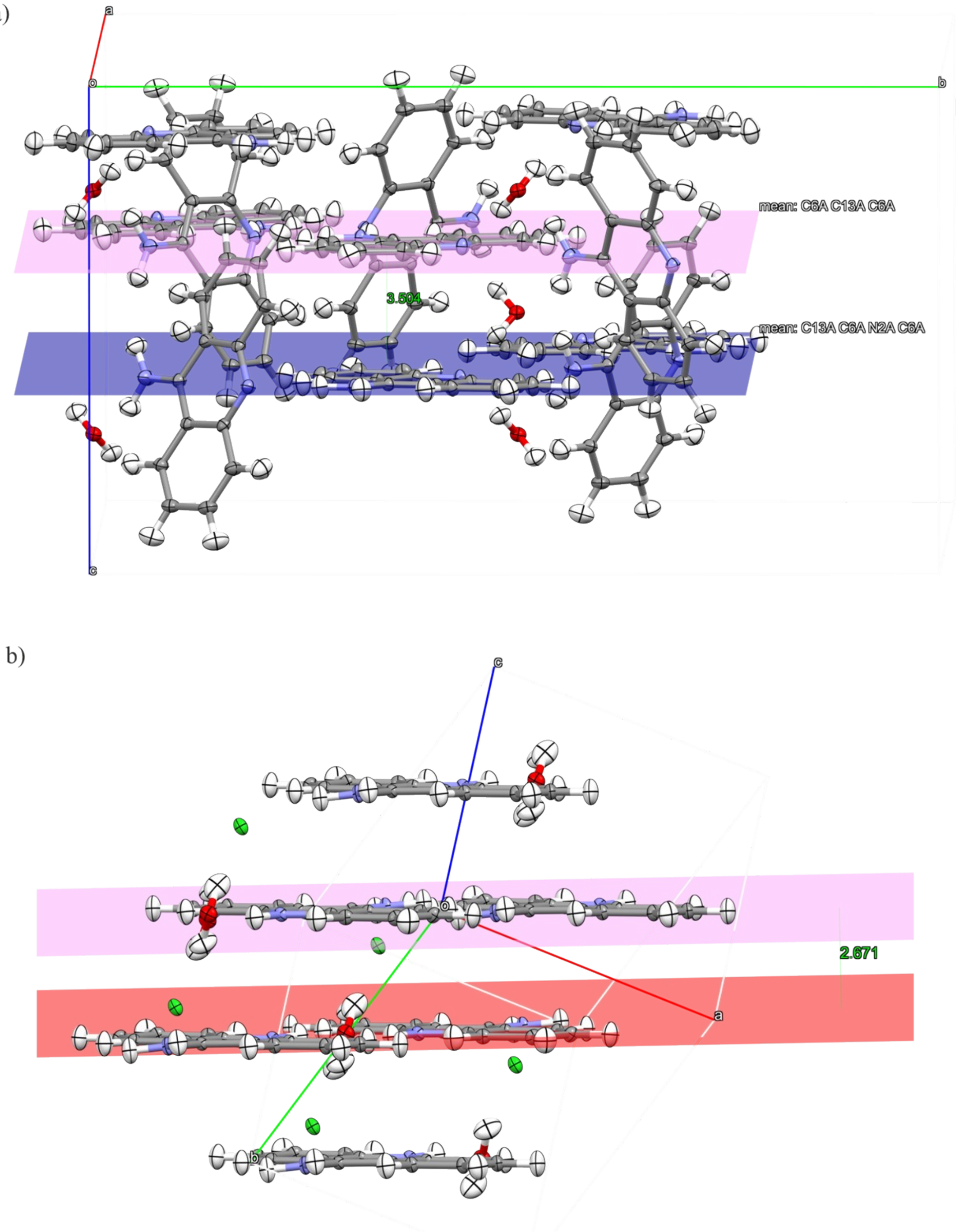

Figure S5. The molecular arrangement of crystal structures of (a) **9aa*H_2O** and (b) **9aa*HCl** with the measured distances between the layers. The distances were measured between the average planes of the subsequent molecular layers defined on the aminacrine rings.

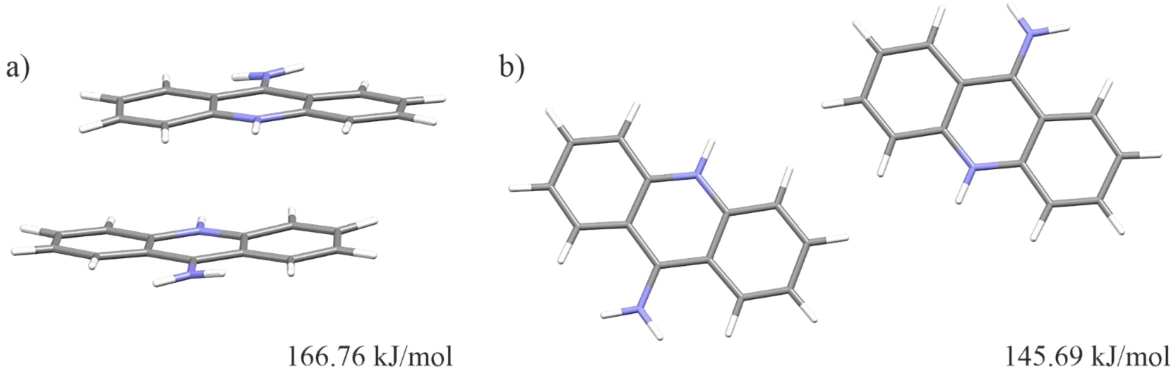

Figure S6. Dimer of interacting molecules for **9aa*HCl** form.

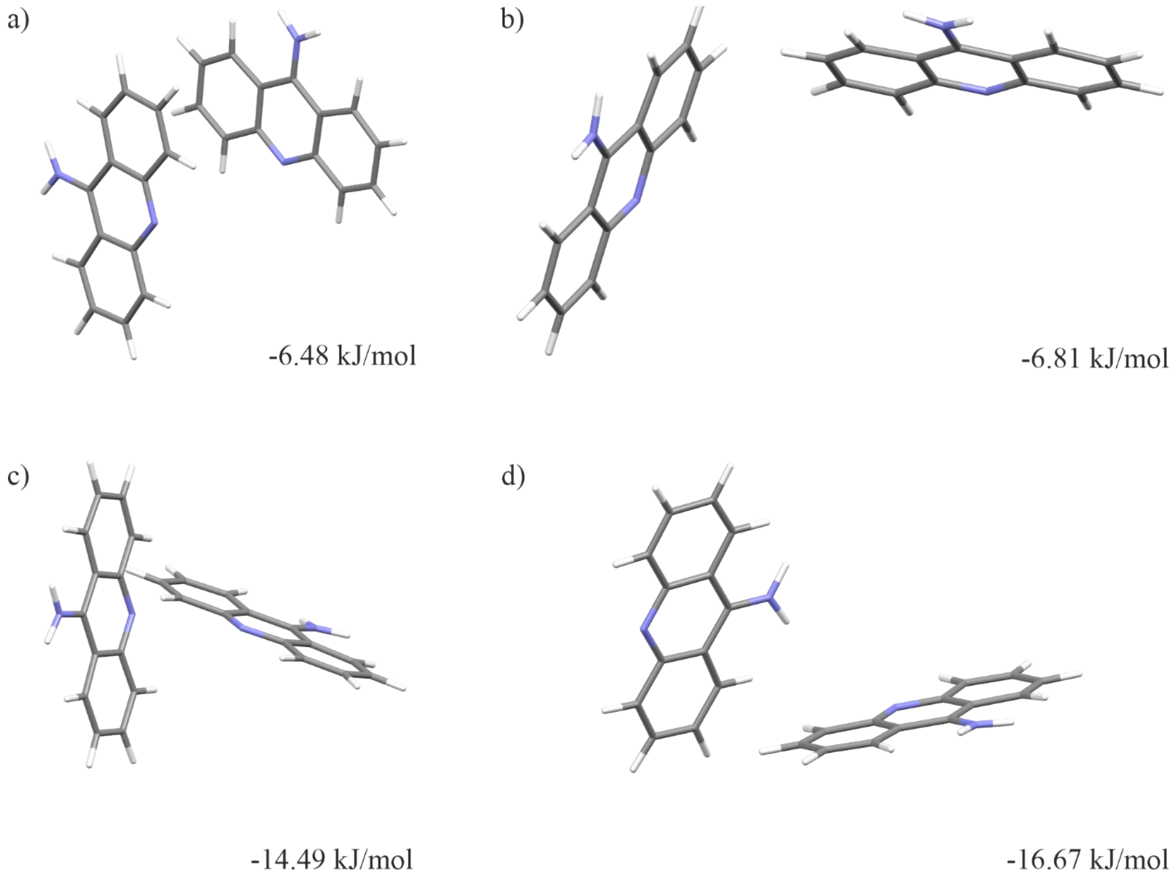

Figure S7. Dimer of interacting molecules for **9aa*H_2O** form.

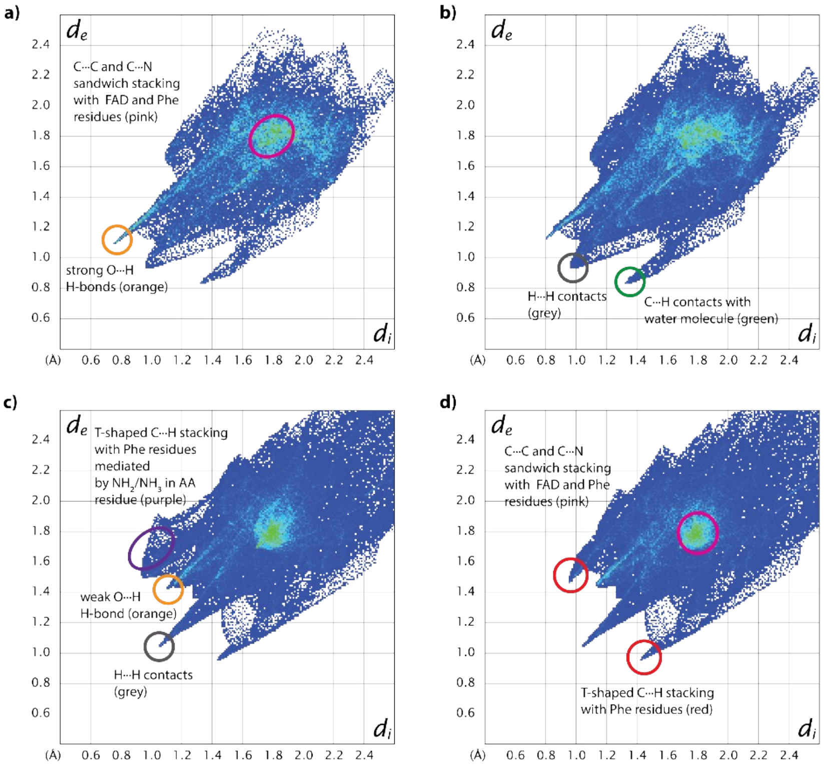

Figure S8. HS fingerprint plots of **9aa** ligands in selected protein structures: 6o4x (human AChE) in neutral (a) or protonated form (b), and 3tzb (quinone oxidoreductase) in neutral (c) and protonated (d).

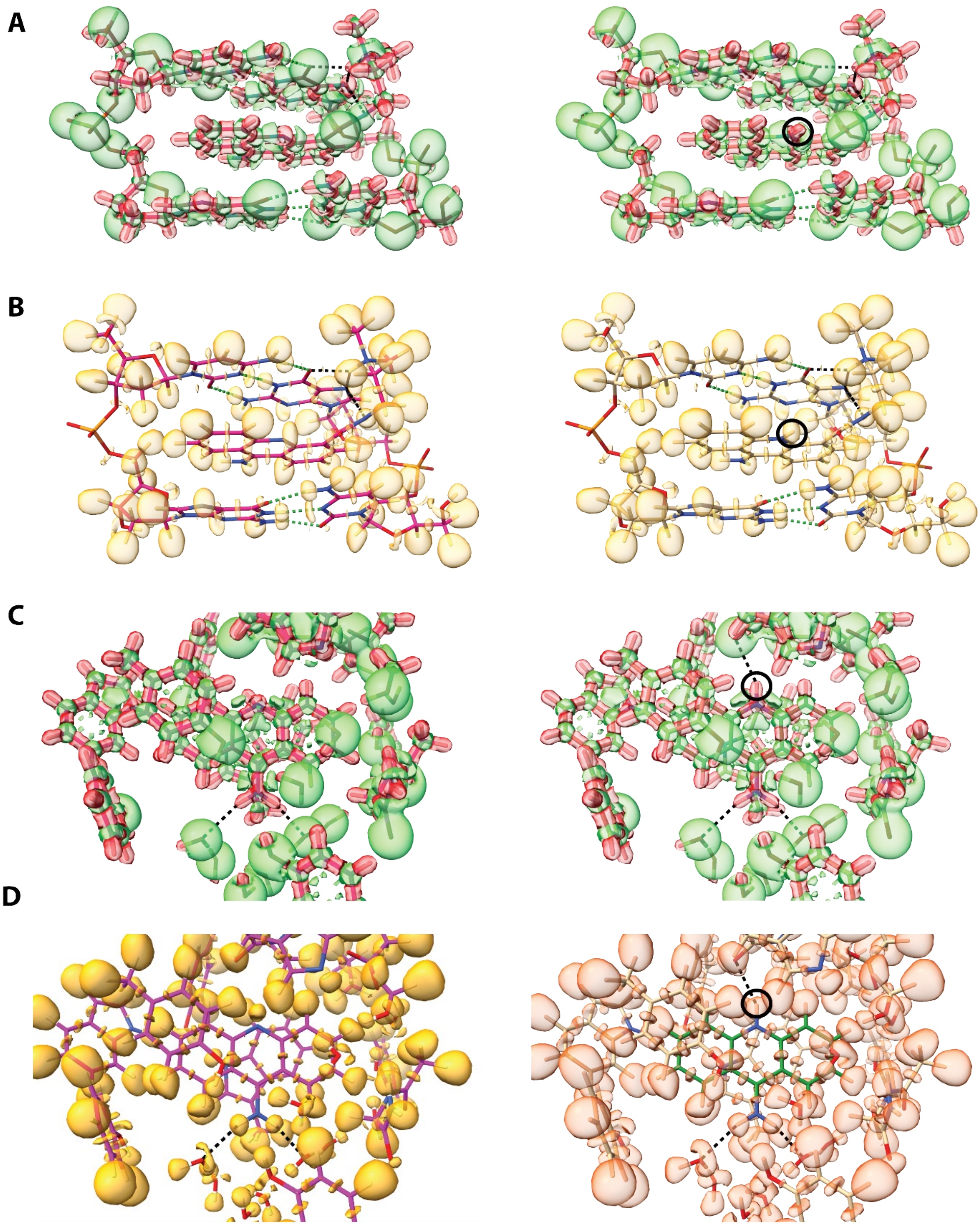

Figure S9. Isosurfaces of electron density functions for macromolecular complexes. Contour levels are -0.23 (red) and +0.23 (green) eA^{-5} for Laplacian and 0.9 for ELF. **A.** ED Laplacian for neutral (left) and protonated (right) form of 9aa complexed by DNA. **B.** ELF for neutral (left) and protonated (right) form of 9aa complexed by DNA. **C.** ED Laplacian for neutral (left) and protonated (right) form of 9aa bound by human acetylcholinesterase. **D.** ELF for neutral (left) and protonated (right) form of 9aa bound by human acetylcholinesterase.

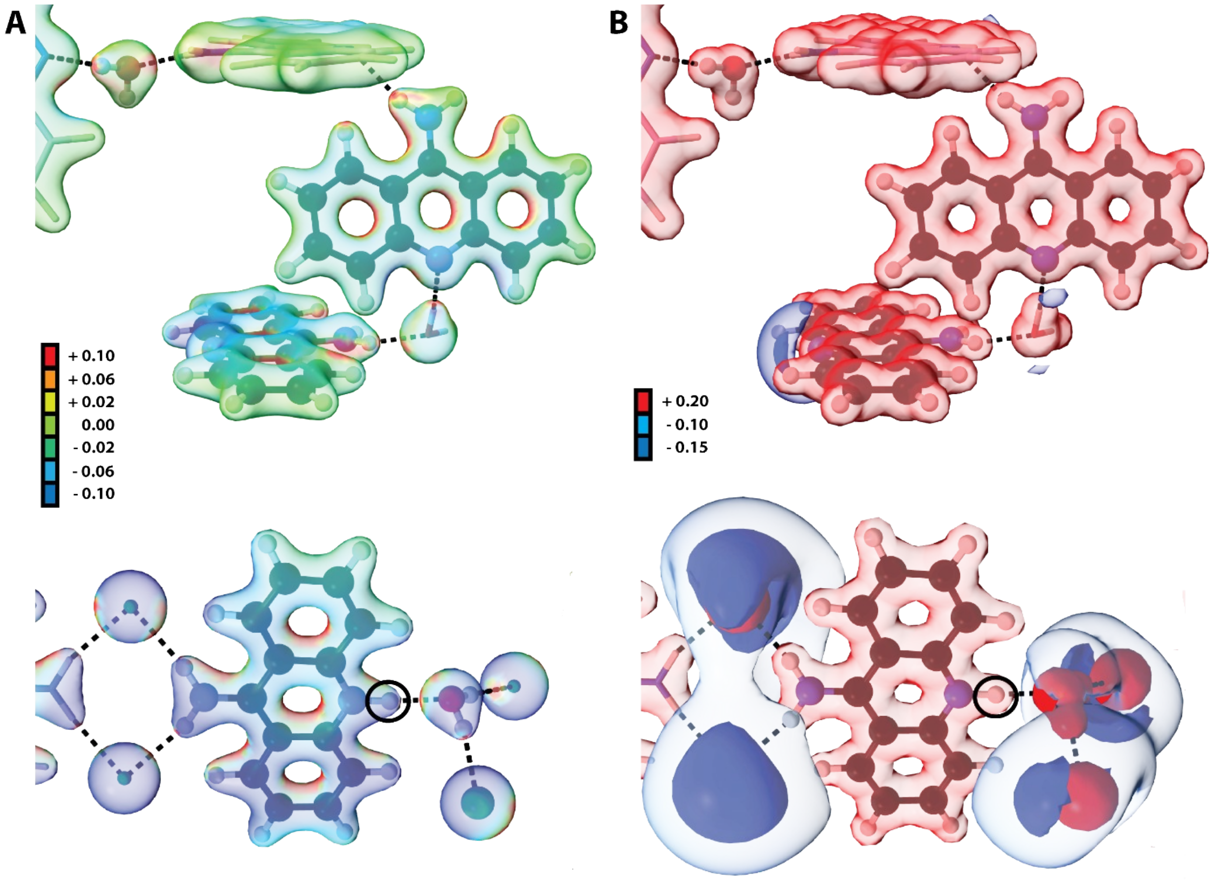

Figure S10. A. Electrostatic potential mapped on isosurfaces of electron density (contour 0.08 eA^{-3}) prepared using rainbow gradient colouring scheme where lowest ESP values are blue and highest are red (-0.1 to +0.1 E_h e^{-1}) for crystal structures of **9aa** in neutral (top) and protonated form (bottom). **B**. Isosurfaces of electrostatic potential at several values for crystal structures of **9aa** in neutral (left) and protonated form (right). For both forms of **9aa** the asymmetric unit is depicted in ball-stick representation and selected adjacent molecules in crystal are depicted as sticks. H-bonds are presented as black dotted lines. Black circles indicate protonation site.

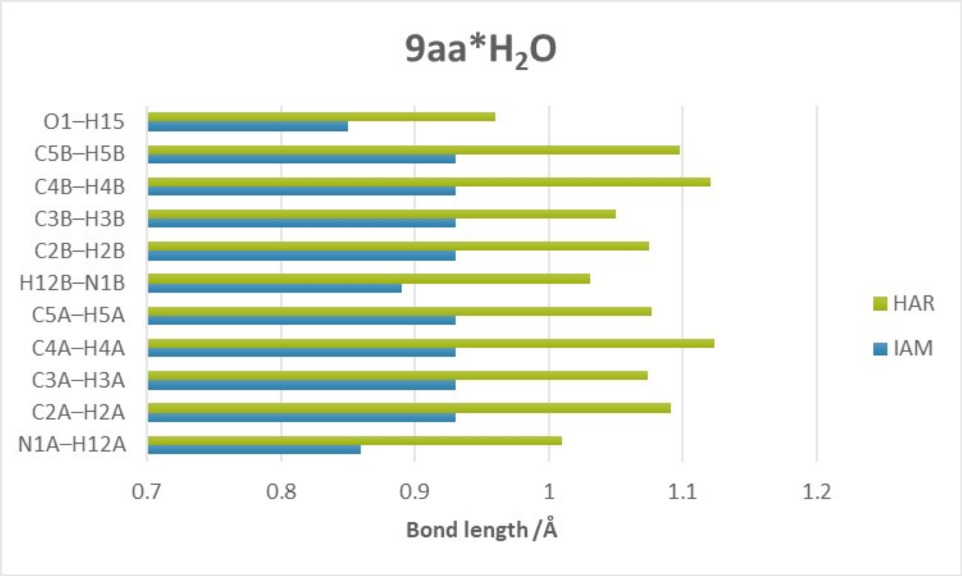

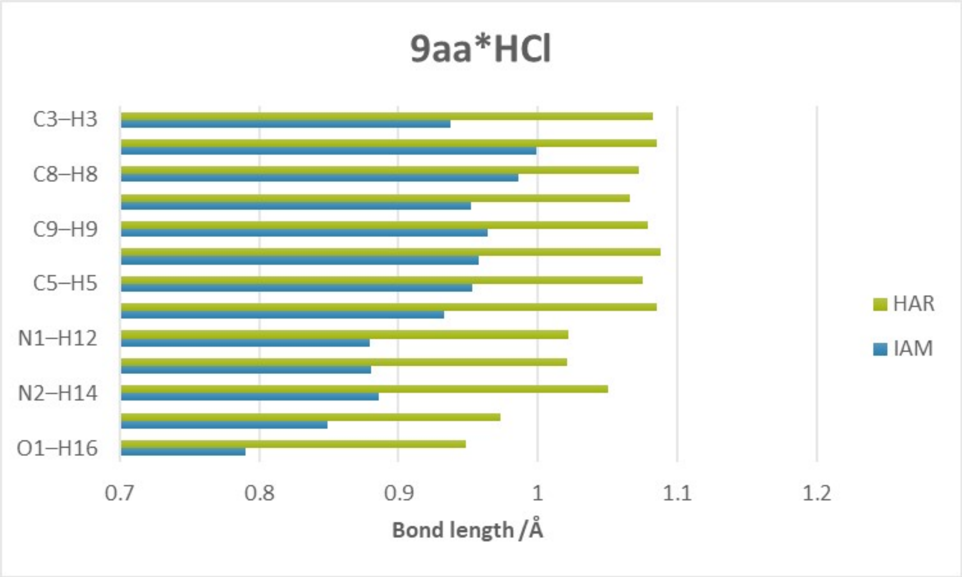

Figure S11. Graph representation of the *X* – H bond lengths obtained from IAM and HAR for both studied forms.
